# The presence of a functional t-tubule network increases the sensitivity of RyR1 to agonists in skinned rat skeletal muscle fibres

**DOI:** 10.1016/j.ceca.2008.02.006

**Published:** 2008-10

**Authors:** Adrian M. Duke, Derek S. Steele

**Affiliations:** Institute of Membrane and Systems Biology, University of Leeds, Woodhouse Lane, Leeds LS29JT, United Kingdom

**Keywords:** Sarcoplasmic reticulum, t-Tubules, RyR1, Ca^2+^ regulation, Skeletal

## Abstract

Single mechanically skinned extensor digitorum longus (EDL) rat fibres were used as a model to study the influence of functional t-tubules on the properties of RyR1 in adult skeletal muscle. Fibres were superfused with solutions approximating to the intracellular milieu. Following skinning, the t-tubules re-seal and repolarise, allowing the sarcoplasmic reticulum (SR) Ca^2+^ release to be activated by field stimulation. However, in the present study, some fibres exhibited localised regions where depolarisation-induced SR Ca^2+^ release was absent, due to failure of the t-tubules to re-seal. When these fibres were exposed to caffeine to directly activate RyR1, regions with re-sealed t-tubules exhibited greater sensitivity to submaximal (2–5 mM) levels of caffeine (*n* = 8), while the response to a supramaximal SR Ca^2+^ release stimulus was uniform (*n* = 8, *p* < 0.05). This difference in RyR1 sensitivity was unaffected by sustained depolarisation of the t-tubule network. However, after saponin permeabilization of the t-tubules or withdrawal of Ca^2+^ from the t-tubules before skinning, the difference in agonist sensitivity was abolished. These results suggest that in adult skeletal muscle fibres, the presence of a functional t-tubule network increases the sensitivity of RyR1 to agonists via a mechanism that involves binding of Ca^2+^ to an extracellular regulatory site.

## Introduction

In skeletal muscle, L-type Ca^2+^ channels (dihydropyridine receptors, DHPRs) located within the t-tubules are functionally coupled to ryanodine receptor Ca^2+^ channels (RyRs), within closely opposed regions of the junctional sarcoplasmic reticulum (SR) [1]. DHPRs are arranged in clusters of four, with each of these “tetrads” linked to a single type1 ryanodine receptor (RyR1), forming a Ca^2+^ release unit (CRU) [2]. The physiological activation process involves t-tubule depolarization, which induces a conformational change involving the α1s II–III loop of the DHPR, leading to activation of RyR1 [1,3,4]. However, CRUs operate as a bidirectional signalling complexes, such that changes in RyR1 gating can provide a retrograde signal that influences DHPR function [5].

A number of previous studies have investigated the relationship between the t-tubule network and RyR1 function in cultured skeletal myotubes. Early experiments on primary cultured mouse myotubes revealed that spontaneous Ca^2+^ sparks occur predominantly in regions of the cell that are unresponsive to depolarisation [6]. It was shown subsequently that mouse myotubes cultured for 72 h have an incomplete t-tubule network, while exhibiting a normal striated RyR1 expression pattern [7]. In these cells, spontaneous Ca^2+^ sparks occur at a much higher frequency in regions lacking t-tubules (i.e. unresponsive to depolarisation), suggesting that the presence of functional t-tubules is associated with suppression of RyR gating. A similar conclusion was reached in a recent study on mouse skeletal fibres, subject to 1–7 days culture and consequent dedifferentiation [8]. The frequency of spontaneous Ca^2+^ sparks was initially low, but increased >10-fold by days 5–7. This marked increase in spark frequency coincided with morphological changes, including disorganization of the t-tubule network, suggesting a causal relationship. Further work revealed that suppression of Ca^2+^ sparks does not occur in dysgenic myotubes (i.e. lacking DHPRs), suggesting that in resting myotubes, the DHPRs within each CRU exert a tonic inhibitory influence on associated RyRs [7].

While these studies on cultured cells suggest a role for the t-tubule/DHPR in the suppression of Ca^2+^ spark activity, it is not clear to what extent these findings translate to adult muscle, where there are clear differences in signalling pathways and the expression levels of key proteins such as RyR3 [9]. This was addressed in a recent study on adult mouse skeletal muscle, where expression of RyR3 resulted in the appearance of abundant Ca^2+^ sparks, while over expression of RyR1 did not [10]. This suggests that in adult skeletal muscle, the paucity of Ca^2+^ sparks can be explained partly by the relatively low level of RyR3 expression, compared with developing myotubes. However, these findings do not exclude the possibility of a tonic inhibitory influence on RyR1 conferred by the t-tubule/DHPR in resting adult muscle.

In the present study, mechanically skinned skeletal fibres from adult rats were used to investigate the influence of functional, polarised t-tubules on RyR1. Following skinning, the t-tubules re-seal and repolarise, allowing the physiological SR Ca^2+^ release mechanism to be initiated by field stimulation. Using confocal microscopy, we showed that a proportion of skinned fibres exhibit localised regions of SR Ca^2+^ release failure, where the t-tubule network failed to re-seal. These fibres were used as a model to compare properties of RyR1 (agonist sensitivity and Ca^2+^ spark frequency) in adjacent regions of the cell with or without a functional t-system.

## Methods

### Preparation

Rats (200–300 g) were humanely killed in accordance with UK legislation (Schedule 1 procedure) Single extensor digitorium longus (EDL) muscle fibres were mechanically skinned under oil and then perfused with solutions approximating to the intracellular *milieu*. Following skinning, the t-tubules re-seal and repolarise, allowing the physiological depolarisation-induced Ca^2+^ release process to be initiated by field stimulation [11].

### Solution composition

All chemicals were purchased from Sigma unless stated otherwise. For most experiments, a basic solution was prepared containing (mM): HDTA, 50; ATP, 8; Na^+^, 37; K^+^, 126; phosphocreatine, 10; EGTA, 0.05; HEPES, 90; fluo-3, 0.02. The free [Mg^2+^] of this solution was adjusted to 1 mM by addition of MgO. The free [Ca^2+^] was ∼60 nM. In most experiments 50 μM *n*-benzyl-*p*-toluene sulphonamide (BTS) was added to inhibit Ca^2+^-activation of the myofilaments [12], thereby minimizing movement artefacts. In control experiments it was found that BTS did not influence SR Ca^2+^ release or the occurrence of localised Ca^2+^ release failure. Corrections for ionic strength, details of pH measurement, allowance for EGTA purity and the principles of the calculations are described elsewhere [13]. All experiments were done at room temperature (20–22 °C), pH 7.1. In most experiments, a maximal SR Ca^2+^ release was induced by rapid addition of a solution with 20 mM caffeine, no added Mg^2+^. However, while the applied solution contains has no added Mg^2+^, there is some mixing with the control solution (free Mg^2+^, 1 mM), which means that the actual [Mg^2+^] at the preparation is ∼20 μM (measured directly using Mg^2+^ fura-2, not shown). Consequently, we have referred to this throughout as application of 20 mM caffeine, low Mg^2+^.

#### Apparatus

The apparatus used for mounting the preparation and application of solutions is described in detail elsewhere [14]. Briefly, the mechanically skinned muscle fibres were mounted in a shallow bath with a coverslip base. The fibres were attached between two fixed supports using monofilament thread (30 μm diameter, Ethicon Ltd., Edinburgh, UK) within stainless steel tubes (inside diameter 100 μm (Goodfellow Cambridge Ltd., Huntington, UK). Platinum electrodes (200 μm) were positioned on either side of the fibre and connected to a stimulator (Grass, model S48). A cylindrical Perspex column (4 mm in diameter) was lowered to within a few microns of the fibre surface. Caffeine was applied rapidly using a purpose built syringe pump. The syringes (5 ml), containing the caffeine solutions, were connected via narrow cannulae (inside diameter, 0.5 mm) to a series of injection ducts near the base of the column. The experimental bath was placed on the stage of a TE3000 Nikon Diaphot inverted microscope. Muscle fibres were viewed via a 40× Fluor objective (Nikon CF Fluor, NA 0.75) and the length was increased to ∼20% above slack length.

#### Detection of SR Ca^2+^ release

Ca^2+^ release from the SR was detected using a Bio-rad Cellmap confocal scanhead attached to the side port of the Nikon TE3000 microscope (excitation 488 nm). In some experiments, sequential frames were collected in *x*–*y* mode before, during and after brief (300–400 ms) tetanic responses initiated by applying suprathreshold square-wave stimuli (70 V cm^−1^, 2 ms duration) at 50 Hz. A relatively slow *x*–*y* frame rate (typically 5–7 Hz) was used to minimize noise on the Ca^2+^ signal. However, the collection rate was sufficient to ensure that one to two frames were obtained during the peak of the peak of each tetanic response.

#### Transport of fluo-3 into the re-sealed t-tubules

We have previously reported that (i) when skinned fibres are bathed in a solution with either fluo-3 or fluo-5N, the dye progressively accumulates within re-sealed t-tubules and associated vesicles and (ii) dye transport into the t-tubules can be inhibited using probenecid [15]. The inhibitory action of probenecid suggests that accumulation of dye by the re-sealed t-tubules occurs via an organic anion transporter, as described in other cell types [16,17]. In some experiments this method was used to load fluo-3 into the re-sealed t-tubules (20 μM, 10 min exposure). As fluo-3 has a relatively high affinity for Ca^2+^, the dye is at or near full saturation within the t-tubules (free [Ca^2+^] is ∼1 mM), allowing regions of the cell with re-sealed t-tubules to be identified using confocal microscopy. In control experiments, this method was compared with an alternative procedure, in which the dye is introduced into the t-tubules before skinning [18]. However, in relation to the phenomena described in this study, there was no apparent difference between the methods.

### Data analysis

Significance (*p* < 0.05) levels were calculated using a Student's *t*-test for unpaired observations and the data are presented as mean ± standard error (S.E.M.).

## Results

**Figure 1:**
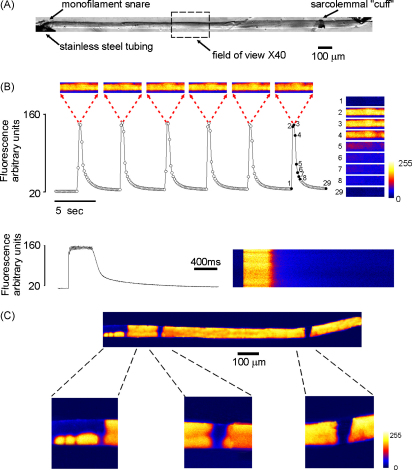
Patchy Ca^2+^ release in mechanically skinned EDL fibres. (A) Composite transmission image of a mechanically skinned EDL muscle fibre produced by taking a series of *x*–*y* frames along its length. In this example, the sarcolemma was not removed along the entire fibre length allowing the “cuff” to be seen. The box (broken line) illustrates the maximum length of fibre observable in a single frame under 40× magnification. (B) Sequential *x*–*y* images (6.2 Hz) from a selected segment of a skinned fibre, stimulated repeatedly at 50 Hz for 400 ms. The graph shows changes in fluo-3 fluorescence, where each point was obtained by averaging the pixels within a single *x*–*y* frame. The individual frames obtained during the peak of each transient show that the tetanic responses are reproducible and that Ca^2+^ release is relatively uniform (*above*). A series of sequential *x*–*y* frames obtained during a single tetanic response is also shown (*right*) and a longitudinal line scan image obtained during a tetanic response (*below*). (C) Composite image showing peak tetanic [Ca^2+^] throughout the length of a mounted EDL fibre. Expanded sections reveal localised regions of Ca^2+^ release failure.

While studies involving force measurements on mechanically skinned skeletal muscle fibres typically use sections 2–3 mm in length, confocal imaging systems allow only a fraction of the preparation to be viewed at one time. Fig. 1A shows a transmission image of a mechanically skinned rat EDL muscle fibre (∼2–3 mm long), tethered at each end using monofilament snares within stainless steel tubes. In this case, an image of the entire mounted fibre was produced by taking a series of *x*–*y* frames along its length, from which a composite image was constructed. The box (broken line) illustrates the maximum length of fibre that could be observed in a single frame under 40× magnification. Although this varies depending on optical arrangement and fibre orientation, this is typically ∼200–300 μm.

Fig. 1B shows recordings from a skinned fibre, which was field-stimulated to produce brief tetanic responses (50 Hz for 400 ms) at ∼5 s intervals, while *x*–*y* images were obtained at 6.2 Hz. This relatively low frame rate optimised the signal-noise-ratio, while ensuring that at least one frame was obtained during the peak of each tetanic response. The graph illustrates the changes in [Ca^2+^] during the period of stimulation, where each point was obtained by integrating a single *x*–*y* frame. The individual *x*–*y* frames obtained during the peak of each transient show that the tetanic responses are reproducible and that Ca^2+^ release is typically uniform in nature. Also shown is a series of sequential *x*–*y* frames obtained during a single tetanic response (*right*) and a transverse line scan image (*below*) obtained during a tetanic response.

While the profile of tetanic [Ca^2+^] was typically uniform in randomly selected regions of each fibre, spatial non-uniformities were observed in approximately 20% of fibres. This is shown in Fig. 1C, where repeated tetanic responses were induced in a skinned fibre and the field of view moved progressively along the cell (as Fig. 1A). The composite image shows the spatial distribution of intracellular Ca^2+^ throughout the fibre, during the peak of the tetanic responses. Pronounced “notches” in the fluorescence profile are apparent, corresponding to regions of the fibre where field stimulation has failed to elicit SR Ca^2+^ release. As shown in the expanded sections, these localised regions of SR Ca^2+^-release failure are variable in size and shape, but have sharply defined boundaries. The pattern of Ca^2+^ release was not altered by changing the polarity of the electrodes, or by increasing the stimulation voltage (*not shown*), suggesting that Ca^2+^ release failure did not reflect an inadequate depolarisation stimulus.

### Regions exhibiting failure of depolarisation-induced Ca^2+^ release lack re-sealed t-tubules

**Figure 2:**
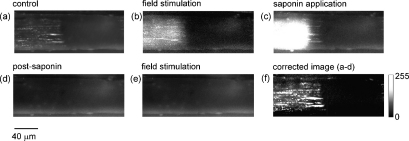
Localised Ca^2+^ release failure occurs where t-tubules have not re-sealed. A skinned EDL fibre was exposed to a solution containing 20 μM fluo-3 acid for 20 min. This resulted in a progressive accumulation of dye within re-sealed t-tubules and vesicular elements on the left of the *x*–*y* image, but not on the right (a). In the continued presence of fluo-3, the preparation was field stimulated (50 Hz for 400 ms) and the resulting rise in cytosolic [Ca^2+^] occurred in a region of the cell exhibiting dye accumulation within the re-sealed t-tubules (b). Application of saponin caused a transient rise in [Ca^2+^] in the same region of the cell (c) and more prolonged exposure (2 min) resulted in loss of dye trapped within the re-sealed t-tubules (d). After saponin treatment, field stimulation failed to elicit a response (e). Subtraction of the fluo-3 fluorescence remaining after saponin treatment (i.e. cytocolic + bound) from the fluorescence in the control image (i.e. cytosolic + bound + t-tubule) provides a corrected image showing the dye localised within the saponin-sensitive compartments (f). All responses were obtained in the same fibre. Corrected image (d) re-scaled to enhance contrast and clearly identify the region with dye trapped in the t-tubule.

The absence of depolarisation-induced SR Ca^2+^ release in localised regions of mechanically skinned fibres (Fig. 1C) might be explained by failure of the t-tubules to re-seal. This possibility was investigated using the protocol shown in Fig. 2, where a skinned fibre was exposed to 20 μM fluo-3 for 20 min, resulting in accumulation of the dye in the t-tubules (see Methods). A region exhibiting a characteristic striated pattern due to t-tubule dye localization [15,19] was positioned on the left of the *x*–*y* frame, while a darker region, apparently lacking trapped dye, occupied the remainder of the field (Fig. 2a). As described in previous studies, the re-sealed region also contained dye trapped within sarcolemmal vesicles, which appear as bright spherical or longitudinal elements [19].

The fact that the t-tubules can be identified despite the continued presence of fluo-3 within the cytosol, suggests that the dye is concentrated within the re-sealed membranes. Field stimulation of the fibre resulted in a rise in [Ca^2+^], detected by the fluo-3 within the cytosolic compartment. The rise in [Ca^2+^] was localised to the region of the cell with dye trapped within the t-tubules (Fig. 2b). Selective permeabilization of the t-tubules with saponin resulted in a rise in [Ca^2+^] due to (1) depolarisation of the t-tubule leading to SR Ca^2+^ release and (2) loss of Ca^2+^ from the t-tubules and vesicular elements (Fig. 2c). The rise of [Ca^2+^] in response to saponin was restricted to the region, which formally exhibited depolarisation-induced Ca^2+^ release and evidence of dye trapped within the t-tubules. The fluorescence image became more uniform and diffuse following saponin treatment and the t-tubule and vesicle structures were no longer apparent (Fig. 2d). Consistent with disruption of the t-tubule membrane, subsequent field stimulation failed to induce Ca^2+^ release from the SR (Fig. 2e). After saponin treatment, the remaining diffuse fluorescence reflects fluo-3 within the cytosol and dye bound to structures within the cell. This remaining fluorescence was subtracted from the overall fluorescence (i.e. Fig. 2a) to provide a corrected image, which represents the dye trapped within the saponin-sensitive membrane compartments (Fig. 2f). Similar results were obtained with four other preparations obtained using the same protocol and in two preparations in which the dye was trapped within the t-tubules before skinning (*not shown*).

### Regions with re-sealed t-tubules exhibit higher sensitivity to RyR1 agonists

**Figure 3:**
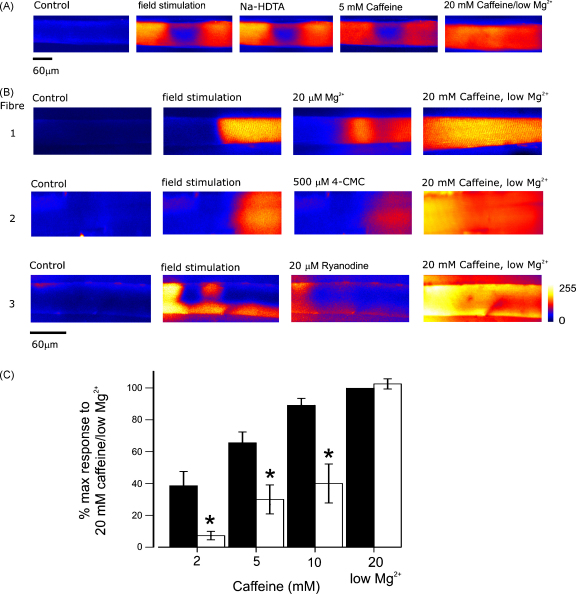
Regions with re-sealed t-tubules have a higher sensitivity to RyR1 agonists. (A) A mechanically skinned fibre was field simulated in order to identify a region exhibiting Ca^2+^ release failure (positioned centrally in the image). Selected *x*–*y* images show the fibre under control conditions and then during the peak of responses to (i) tetanic stimulation (50 Hz for 400 ms), (ii) depolarisation of the t-tubules by substitution of K-HDTA with Na-HDTA, (iii) addition of 5 mM caffeine and (iv) 20 mM caffeine/low Mg^2+^. (B) Following field stimulation (50 Hz for 400 ms) to identify regions Ca^2+^ release failure, fibres 1–3 were exposed to a variety of stimuli, which induce a submaximal Ca^2+^ efflux from the SR by activating RyR1 including (1) 20 μM Mg^2+^, (2) 500 μM 4-CMC, or (3) 20 μM ryanodine. In each case, the submaximal stimulus was followed by a maximal response to 20 mM caffeine/low Mg^2+^. (C) Accumulated data illustrating the difference in caffeine sensitivity of regions responsive (filled bars) or un-responsive (open bars) to field stimulation. All responses are expressed relative to that induced by application of 20 mM caffeine/low Mg^2+^ in the responsive region. (*) Indicates statistically different from response in region exhibiting depolarisation-induced Ca^2+^ release (*p* < 0.05, *n* = 5, mean ± S.E.M.).

The presence of readily identifiable regions of skinned fibres lacking re-sealed t-tubules was used to study the functional relationship between the DHPR/t-tubule network and associated RyRs located within the junctional SR. In Fig. 3, fibres were repeatedly field-stimulated at 50 Hz (400 ms duration) in order to identify a localised region exhibiting failure of depolarisation-induced SR Ca^2+^ release (not shown). In A, the Ca^2+^ release profile during the peak of a tetanic response to field stimulation is shown adjacent to responses induced by substitution of K-HDTA with Na-HDTA, addition of a submaximal concentration of caffeine (5 mM) or maximal activation of RyR1 induced by 20 mM caffeine/low Mg^2+^ (see Methods). As expected, the Ca^2+^-release profile in response to depolarisation of the t-tubule network following Na^+^ substitution was similar to that induced by field stimulation. In this cell, 2 mM caffeine failed to induce a response (not shown). A pronounced SR Ca^2+^ release did occur on introduction of 5 mM caffeine, but only in the regions responsive to t-tubule depolarisation. This suggests that the sensitivity of RyR1 is higher in regions of the fibre were RyRs are associated with re-sealed t-tubules. Subsequent addition of 20 mM caffeine/low Mg^2+^ to induce maximal activation of RyR1, resulted in a spatially homogeneous rise in [Ca^2+^]. Control experiments showed that the dye was not saturated during a maximal SR Ca^2+^ release.

Fig. 3B shows that the differential sensitivity of RyR1 in regions of the fibre segment where the t-tubules are sealed or un-sealed is also apparent with a range of other factors inducing submaximal SR Ca^2+^ release including (1) a decrease in free [Mg^2+^] to 20 μM, (2) 500 μM 4-CMC or (3) 20 μM ryanodine. In each fibre, although each intervention released less Ca^2+^ than t-tubule depolarisation, the spatial distribution of Ca^2+^ was similar. Fig. 3C shows accumulated data illustrating the relative caffeine sensitivity of neighbouring regions, which were either responsive or unresponsive to field stimulation.

### Differential sensitivity to RyR2 agonists is unrelated to t-tubule polarisation

As considered above, regions of the cell in which the t-tubule network has apparently failed to re-seal following skinning, exhibit a lower sensitivity to RyR1 agonists. This might reflect the polarization status of the t-tubule membrane in re-sealed regions, e.g. (1) if the configuration of the DHPRs in regions of the cell with polarised t-tubules differs from that in regions with un-sealed (depolarised) t-tubules, such that a higher sensitivity to RyR1 agonists is conferred or (2) if re-sealed t-tubules are subject to spontaneous, subthreshold fluctuations in membrane potential, which leads to a reduced inhibitory influence of the DHPRs on associated RyRs.

**Figure 4:**
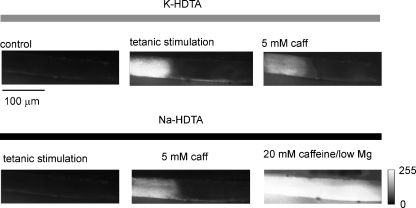
Patchy Ca^2+^ release persists after depolarisation of the t-tubules. A mechanically skinned fibre was initially perfused with a solution containing K-HDTA and field-stimulated in order to identify a region exhibiting SR Ca^2+^ release failure (*upper panel*). Selected *x*–*y* images are shown under control conditions (*left*) and during the peaks of responses to tetanic field stimulation (*middle*) or 5 mM caffeine (*right*). The same fibre was then exposed to a solution in which K-HDTA was substituted with Na-HDTA to induce a sustained depolarisation of the t-tubules (*lower panel*). The initial exposure to Na-HDTA induced a localised rise in Ca^2+^ due to depolarisation of the t-tubule network (not shown). In the continued presence of Na-HDTA, field stimulation failed to elicit a response (*left*). On addition of 5 mM caffeine, the rise in [Ca^2+^] occurred in the region of the cell responsive to field stimulation (*middle*). Subsequent addition of 20 mM caffeine/low Mg^2+^ induced a uniform Ca^2+^ release.

The role of t-tubule polarisation was investigated using the protocol shown in Fig. 4. A fibre was initially field-stimulated and regions lacking SR Ca^2+^ release identified (*upper panel*). As in previous examples, only the region exhibiting depolarisation-induced Ca^2+^ release (*middle*) responded to 5 mM caffeine (*right*). The same fibre was then perfused with a solution in which K-HDTA was substituted with Na-HDTA in order to induce sustained depolarisation of the t-tubules (*lower panel*). Following Na-substitution, the fibre was unresponsive to field stimulation, consistent with sustained depolarisation of the t-tubule network (lower, *left*). However, on subsequent addition of 5 mM caffeine, the pattern of Ca^2+^ release remained similar to that observed under control conditions (*lower*, *middle*). This suggests that the higher sensitivity to caffeine in regions responsive to field stimulation is unrelated to the polarisation status of the re-sealed t-tubules. Again, addition of 20 mM caffeine/low Mg^2+^ induced a uniform release throughout the fibre (*lower*, *right*), demonstrating that RyRs remain functional throughout the preparation and that the SR [Ca^2+^] content is relatively uniform. Similar results were obtained in five other preparations.

### T-tubule permeabilization confers uniform Ca^2+^ release

**Figure 5:**
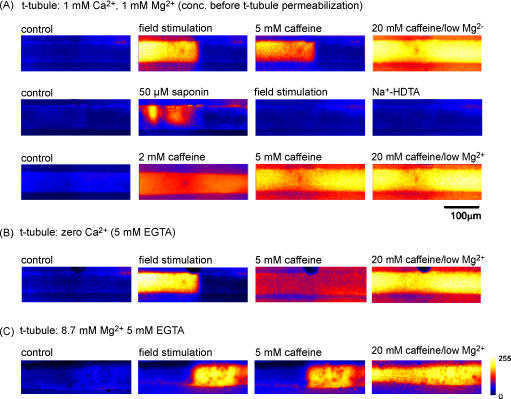
Effects of t-tubule permeabilization or Ca^2+^ depletion on SR Ca^2+^ release. (A) A mechanically skinned fibre was field-stimulated to identify a region exhibiting SR Ca^2+^ release failure. Selected *x*–*y* images are shown under control conditions and during the peak of responses to, tetanic field stimulation, 5 mM caffeine or 20 mM caffeine/low Mg^2+^ (*upper panel*). The same preparation was then exposed to 50 μg/ml saponin, which resulted in a localised rise in [Ca^2+^] due to permeabilization and depolarisation of the re-sealed t-tubules. After saponin treatment, neither field stimulation nor substitution of K-HDTA with Na-HDTA elicited a response, consistent with irreversible disruption of the t-tubule network (*middle panel*). After saponin treatment, both submaximal responses to 2 or 5 mM caffeine and the maximal response to 20 mM caffeine/low Mg^2+^ were spatially uniform (*lower panel*). Similar results were obtained in four other cells. (B) A fibre was mechanically skinned in the presence of 5 mM EGTA in order to reduce the [Ca^2+^] within the t-tubule network to nanomolar levels. Following skinning, field-stimulation was used to identify localised regions of SR Ca^2+^ release failure. On addition of 5 mM caffeine, the resulting Ca^2+^ release was uniform. Similar results were obtained in four other cells. (C) A fibre was exposed to Tyrode's solution, containing 10 mM total Mg^2+^ (8.7 mM free) and 5 mM EGTA before skinning. Field stimulation was again used to identify localised Ca^2+^ release failure. In the presence of 8.7 mM t-tubule Mg^2+^, localised regions exhibiting Ca^2+^ release failure were apparent, and regions exhibiting SR Ca^2+^ release in response to field stimulation were more sensitive to caffeine. Similar results were obtained in four other cells.

In the experiment shown in Fig. 5A, a skinned fibre was field stimulated, allowing a localised region lacking SR Ca^2+^ release to be identified. As in previous examples, addition of 5 mM caffeine induced Ca^2+^ release only in the region sensitive to field-stimulation, while 20 mM caffeine/low Mg^2+^ induced a uniform SR Ca^2+^ release throughout the preparation (*upper panel*). The preparation was then returned to a control solution before exposure to 50 μg/ml saponin for 10 min (*middle panel*). The initial exposure to saponin induced a detectable release of Ca^2+^ due to selective permeabilization (and depolarisation) of the re-sealed t-tubule network. Following removal of saponin, neither substitution of K-HDTA with Na-HDTA nor field stimulation induced a detectable rise in [Ca^2+^], consistent with permeabilization of the t-tubule network. However, after exposure to saponin, submaximal concentrations of caffeine (2 or 5 mM) induced a uniform release of Ca^2+^ throughout the fibre (*lower panel*). Exposure to 20 mM caffeine/low Mg^2+^ induced a maximal release of Ca^2+^, which was comparable to that obtained prior to saponin treatment, suggesting that saponin treatment did not affect the SR Ca^2+^ content or the distribution of Ca^2+^ within the SR network. Similar results were obtained in four other preparations.

A possible explanation for the uniform Ca^2+^ release following exposure to saponin is that the responsiveness of RyR1 may be influenced by the presence of Ca^2+^ within the re-sealed t-tubule, which is lost on permeabilization. Therefore, a further series of experiments was carried out in which cells were initially exposed to a Ca^2+^-free Tyrode's solution (containing 5 mM EGTA). Following skinning, the free [Ca^2+^] within the t-tubule was calculated to be ∼1 nM (based on 10 μM contamination), and the presence of millimolar EGTA within the t-tubules ensured that the [Ca^2+^] remained low for a prolonged period, despite any slow accumulation of Ca^2+^ from the bathing solution. In most of these preparations, field stimulation continued to release substantial Ca^2+^ from the SR, allowing identification of localised regions exhibiting Ca^2+^ release failure (Fig. 5B). However, in the effective absence of t-tubule Ca^2+^, addition of submaximal levels of caffeine typically induced a *uniform* release of Ca^2+^ from the SR. Similar results were seen in four other preparations.

In a further set of experiments, cells were exposed to Tyrode's solution, containing 10 mM total Mg^2+^ (8.7 mM free) and 5 mM EGTA, before skinning (Fig. 5C). Field stimulation was again used to identify localised Ca^2+^ release failure. However, in the presence of millimolar t-tubule Mg^2+^, the differential sensitivity to caffeine was sustained. Similar results were obtained in four other cells.

### Properties of Ca^2+^ sparks in regions of the cell with and without functional t-tubules

In intact adult mammalian fibres, spontaneous, localised Ca^2+^ release events are only observed at high frequency following membrane stress or damage [20]. However, Ca^2+^ sparks or embers can be detected in skinned or permeabilized fibres [21], possibly due to changes in mitochondrial redox potential [22]. While, the physiological relevance of these phenomena remain uncertain, the properties of sparks and embers were characterised in regions with or without re-sealed t-tubules to allow comparison with previous studies on cultured cells.

**Figure 6:**
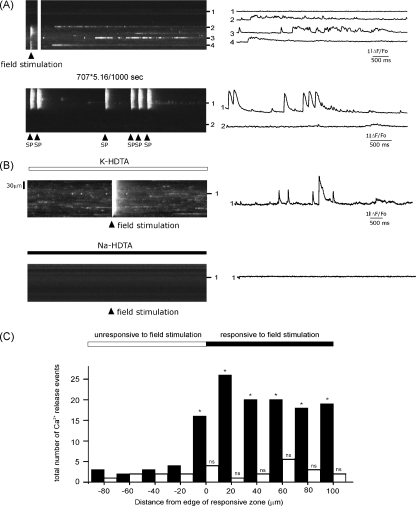
Spontaneous localised Ca^2+^ release events in sealed and unsealed regions. (A) Longitudinal line scan images from two mechanically skinned fibres. In the first cell, approximately half of the scan line was positioned in a region responsive to field stimulation, while the other half was positioned in an adjacent unresponsive region (upper panel). Ca^2+^ sparks and more prolonged “embers” are apparent, but most are restricted to the responsive region. A fibre exhibiting spontaneous SR Ca^2+^ release events is also shown (lower panel). Again, spontaneous Ca^2+^ release events are restricted to the region exhibiting spontaneous release events. (B) Longitudinal line scan images from a mechanically skinned fibre in the presence of either K-HDTA (upper panel) or Na-HDTA (lower panel). In the presence of K-HDTA spontaneous Ca^2+^ release events are apparent and the Ca^2+^ transient in response to field stimulation confirms that the t-tubules are polarised and functional throughout the image. On substitution of Na-HDTA with K-HDTA, both the spontaneous Ca^2+^ release events and the response to field stimulation were abolished. (C) Cumulative data obtained from four fibres exhibiting localised Ca^2+^ release events. In each cell, half of the longitudinal scan line was positioned in a region responsive to field stimulation and the other half in a region unresponsive to field stimulation. The ordinate shows the total number of spontaneous Ca^2+^ release events in four fibres at the position indicated either before (black bars) or after depolarisation of the t-tubule network with Na-HDTA (open bars). All distances are relative to the boundary between adjacent responsive and unresponsive regions of the cell. (*) Indicates significantly greater number of events than in adjacent unresponsive region (*p* < 0.05, *n* = 4, mean ± S.E.M.). ns indicates not significantly different from adjacent unresponsive region.

Fig. 6A, shows a recording in line-scan mode from a mechanically skinned fibre, with the scan line positioned longitudinally through adjacent regions, which were either responsive, or non-responsive to field stimulation. A single 2 ms stimulus resulted in a brief twitch response and a corresponding rise in cytosolic Ca^2+^, which was limited to the area of the cell in the lower half of the image (*left*). While quiescent, the cell also exhibited Ca^2+^ sparks and longer embers, similar to those reported in intact and skinned skeletal cells. However, these events were predominantly restricted to the region of the cell responsive to field stimulation. As described previously [11], in the absence of cytocolic Cl^−^, which normally serves to stabilize the membrane potential, some skinned cells exhibit spontaneous depolarisations, resulting in repeated twitch-like responses. An example of this is shown in Fig. 6B, where large Ca^2+^ transients arose spontaneously at irregular intervals. In this case the upper half of the scan line was positioned through a region producing spontaneous Ca^2+^ transients, while the lower half was quiescent and insensitive to field stimulation (*not shown*). Again, spontaneous Ca^2+^ sparks were apparent, but only at high frequency in the region of the fibre exhibiting spontaneous Ca^2+^ transients.

Fig. 6B shows a line scan image from a region of a fibre which exhibiting spontaneous Ca^2+^ release events (upper panel). Field stimulation resulted in a homogeneous rise in [Ca^2+^] throughout the fibre, indicating that the presence of functional, polarised t-tubules throughout. The fibre was then exposed to a Na-HDTA solution to induce sustained depolarisation of the t-tubule network. Thereafter, both spontaneous Ca^2+^ release events were markedly reduced in frequency and field stimulation failed to elicit a response. Fig. 6C shows cumulative data obtained from four fibres exhibiting localised Ca^2+^ release events. In each cell, half of the longitudinal scan line was positioned in a region responsive to field stimulation and the other half in an unresponsive region. The ordinate shows the total number of spontaneous Ca^2+^ release events in four fibres at the position indicated either before (black bars) or after depolarisation of the t-tubule network with Na-HDTA (open bars). All distances are relative to the boundary between responsive and unresponsive regions of the cell. The data show that in cells exhibiting spontaneous Ca^2+^ sparks and embers in regions responsive to field stimulation, sustained depolarisation of the t-tubule membrane reduces the frequency of spontaneous Ca^2+^ release events to a low level, which is not significantly different to that in the unresponsive regions.

## Discussion

In this study we have shown that a proportion of mechanically skinned fibres exhibit localised regions in which field stimulation fails to induce SR Ca^2+^ release (Fig. 1). This SR Ca^2+^ release failure occurs in areas of the cell in which the t-tubules have not fully re-sealed following removal of the sarcolemma (Fig. 2). The main finding was that in regions of fibres with functional, re-sealed t-tubules, the sensitivity to caffeine was higher than in adjacent regions where the t-tubules had failed to re-seal (Fig. 3). This effect was also apparent with a range of other submaximal stimuli, which increase the open probability of the RyR1, including 4-CMC, ryanodine and [Mg^2+^] depletion (Fig. 3). Localised regions of Ca^2+^ release failure have not been reported previously in skinned fibres. However, in experiments involving force measurement, such fibres are likely to be rejected because localised Ca^2+^ release failure will result in a compliant region, which will reduce overall recorded force.

### Possible mechanisms underlying increased agonist sensitivity in regions with re-sealed t-tubules

The response to a stimulus inducing a maximal SR Ca^2+^ release (20 mM caffeine/low Mg^2+^) was similar in regions with or without re-sealed t-tubules (Fig. 3B). This suggests that the difference in sensitivity to factors inducing submaximal RyR1 activation cannot be explained by regional differences in the SR Ca^2+^ content. The difference in sensitivity to caffeine was also unaffected by substitution of K-HDTA with Na-HDTA (Fig. 4), confirming that the polarisation status of the t-tubule is unrelated to the higher RyR1 agonist sensitivity in regions with re-sealed t-tubules.

In fibres exhibiting a higher sensitivity to submaximal levels of caffeine in regions with re-sealed t-tubules, selective permeabilization of the sarcolemmal membrane resulted in a uniform Ca^2+^ release profile (Fig. 5A). A similar uniform response was obtained when intact cells were exposed to solutions with EGTA and no added Ca^2+^ prior to skinning, thereby ensuring that the [Ca^2+^] within the t-tubule lumen was in the nanomolar range following re-sealing. Together, these observations suggest that the presence of extracellular Ca^2+^ underlies or contributes to the higher RyR1 agonist sensitivity apparent in regions with re-sealed t-tubules.

There are a number of possible mechanisms by which t-tubule (i.e. extracellular) Ca^2+^ might exert an influence on RyR1, e.g. it has been shown that in some circumstances, depletion of the SR Ca^2+^ leads to activation of a sarcolemmal Ca^2+^ influx pathway (store operated Ca^2+^ entry, SOCE) [23]. In skinned cells, this can be detected as a depletion of t-tubule Ca^2+^ following activation of RyR1 [18]. Hence, activation of this pathway in regions with re-sealed t-tubules might amplify or otherwise influence the rise in [Ca^2+^] that occurs in the vicinity of the RyR on application of caffeine. However, in control experiments using fluo-5N to measure Ca^2+^ within the t-tubules, submaximal levels of caffeine (≤5 mM applied with 1 mM Mg^2+^) did not induce sufficient SR Ca^2+^ depletion to activate SOCE (*n* = 4, not shown). Furthermore, we show that Mg^2+^ can replace Ca^2+^ in the t-tubule network and confer higher agonist sensitivity in regions with re-sealed t-tubules (Fig. 5C). As Mg^2+^ inhibits RyR1 activation [24], this also suggests that influx of Ca^2+^ from the re-sealed t-tubule network does not explain the higher agonist sensitivity.

An alternative possibility is simply that the binding of extracellular Ca^2+^ to the DHPR results in increased agonist sensitivity of the RyR1 a via a long-range allosteric mechanism. Early studies addressing the role of extracellular Ca^2+^ in excitation contraction coupling are consistent with this potential mechanism: *First*, although Ca^2+^ entry is not essential for excitation–contraction coupling in skeletal muscle, the binding of extracellular Ca^2+^ to the DHPR is required for normal activation and other divalent ions can substitute for Ca^2+^ at this site [25–27]. *Second*, studies on intact skeletal preparations from normal muscle and muscle susceptible to malignant hyperthermia have shown that withdrawal of extra Ca^2+^ reduces the sensitivity of RyR1 to caffeine [28]. Importantly, this study also showed that the caffeine response could be restored by inclusion of millimolar Sr^2+^ or other divalent ions in the extracellular solution, consistent with involvement of an extracellular divalent binding site. *Third*, while little trans-sarcolemmal Ca^2+^ entry occurs during muscle activation, fibres from malignant hyperthermia susceptible muscle often fail to respond to triggering levels of halothane in the absence of extracellular Ca^2+^ [29]. As in the present study, these findings suggest that the binding of extracellular Ca^2+^ is associated with a conformational state of the DHPR, which increases the agonist sensitivity of the RyR1.

### Ca^2+^ sparks arise predominantly in regions with re-sealed t-tubules

In approximately 10% of cells, spontaneous Ca^2+^ sparks arose spontaneously or could be induced by a brief pulse of Na-HDTA to induce partial depolarisation of the t-tubule network (Fig. 6). These events are comparable to those reported previously in skinned fibres [21]. In cells exhibiting localised SR Ca^2+^ release failure, the frequency of spontaneous sparks and embers was markedly higher in regions with re-sealed t-tubules. However, sustained depolarisation of the t-tubules reduced the frequency of spontaneous Ca^2+^ sparks to a level that was not significantly different from that in regions lacking re-sealed t-tubules (Fig. 6). This suggests that the underlying mechanism may involve fluctuations in the membrane potential of the re-sealed t-tubules. Mechanically skinned cells are prone to spontaneous depolarisation of the t-tubule network [11], and although such events can induce substantial intracellular Ca^2+^ transients (Fig. 6B), it seems likely that subthreshold depolarisations also occur. This is particularly the case in the absence of Cl^−^ (generally omitted in skinned fibre studies), which normally serves to stabilize the membrane potential in intact cells [30]. Hence, as with partial depolarisation of the t-tubule membrane in experiments utilizing the Vaseline gap technique [31], sparks may arise due to voltage dependent changes in the configuration of the DHPR. It is important to emphasise that this mechanism differs from that underlying the higher agonist sensitivity in regions with re-sealed t-tubules as this phenomenon was unaffected by sustained depolarisation of the t-tubules (Fig. 4).

### Conclusion and physiological relevance

The present study demonstrates that extracellular divalent ions influence the gating properties of RyR1 and its sensitivity to activation by pharmacological agents. This finding may have relevance to studies on muscle from patients with malignant hyperthermia, where direct activation of RyR1 by triggering anaesthetics does not occur in the absence of extracellular Ca^2+^ [29]. Similarly, it has been found that in aged murine skeletal muscle, the decline in force (and intracellular tetanic [Ca^2+^]) during fatiguing stimulation is dependent upon the presence of extracellular Ca^2+^ [32]. This might be explained by activation of a store operated Ca^2+^ entry pathway [33]. However, the present study introduces the additional possibility that extracellular Ca^2+^ may affect the development of fatigue by acting on RyR1 indirectly via, a mechanism that involves occupation of an extracellular divalent ion binding site, most likely associated with the DHPR.
